# A Totally Laparoscopic Transhiatal Approach for Esophageal Cancer During Mediastinoscopic Esophagectomy

**DOI:** 10.3390/cancers18193236

**Published:** 2026-10-08

**Authors:** Hirotaka Konishi, Shutaro Sumiyoshi, Hiroyuki Inoue, Keiji Nishibeppu, Toshiyuki Kosuga, Yusuke Yamamoto, Ryo Morimura, Hitoshi Fujiwara, Atsushi Shiozaki

**Affiliations:** Division of Digestive Surgery, Department of Surgery, Kyoto Prefectural University of Medicine, Kajii-cho 465, Kamigyo-ku, Kyoto City 6028566, Japan; s-sumi@koto.kpu-m.ac.jp (S.S.); inoue-h@koto.kpu-m.ac.jp (H.I.); nishibe@koto.kpu-m.ac.jp (K.N.); toti-k@koto.kpu-m.ac.jp (T.K.); yamayu-1@koto.kpu-m.ac.jp (Y.Y.); morimura@koto.kpu-m.ac.jp (R.M.); hfuji@koto.kpu-m.ac.jp (H.F.); shiozaki@koto.kpu-m.ac.jp (A.S.)

**Keywords:** mediastinoscopic esophagectomy, laparoscopy, transhiatal approach

## Abstract

Background: Mediastinoscopic esophagectomy is a minimally invasive approach that avoids thoracotomy and single-lung ventilation. We previously performed this procedure using hand-assisted laparoscopic surgery (HALS) and recently established a totally laparoscopic transhiatal approach. This retrospective, non-randomized study reported laparoscopic technique and compared the feasibility and surgical outcomes of these approaches. Methods: We retrospectively reviewed 262 patients who underwent radical mediastinoscopic esophagectomy using left cervical and transhiatal approaches at the Kyoto Prefectural University of Medicine between January 2020 and June 2026. HALS was performed in 202 patients and laparoscopy in 60 patients. Surgical procedures and perioperative outcomes were compared between the groups. Results: Blood loss and total operative time did not differ significantly between the groups, although the transhiatal procedure tended to require a longer operative time with laparoscopy (*p* = 0.09). The approach used for middle mediastinal lymphadenectomy was similar between the two groups (43% vs. 35%, *p* = 0.28), and the overall number of dissected lymph nodes, excluding the upper mediastinal and cervical nodes, was comparable. However, significantly more abdominal lymph nodes were dissected with laparoscopy than with HALS (17.9 vs. 15.7, *p* = 0.04). Laparoscopic gastric mobilization and lymph node dissection were safely performed, with no significant differences in the rate of postoperative complications or curability. Conclusions: The totally laparoscopic transhiatal technique is a feasible and safe approach for mediastinoscopic esophagectomy, with surgical outcomes comparable to those of HALS and not inferior abdominal lymph node dissection. It may be particularly advantageous for cases requiring meticulous abdominal lymphadenectomy.

## Simple Summary

This retrospective, non-randomized study compared hand-assisted laparoscopic surgery (HALS) with a totally laparoscopic approach during the transhiatal phase of mediastinoscopic esophagectomy. We reviewed 262 patients who underwent radical mediastinoscopic esophagectomy using left cervical and transhiatal approaches between 2020 and 2026. HALS was performed in 202 patients, and laparoscopy in 60 patients. Operative time and blood loss were comparable between the groups. The extent of mediastinal lymphadenectomy was similar between the approaches, whereas significantly more abdominal lymph nodes were dissected with laparoscopy than with HALS (17.9 vs. 15.7, *p* = 0.04). Laparoscopy enables safe gastric mobilization and lymph node dissection. Although HALS facilitates instrument stabilization, gastric manipulation, and liver retraction, the surgeon’s hand may interfere with the dissection along the superior border of the pancreas. Therefore, laparoscopy may be particularly useful in cases requiring more precise abdominal lymph node dissection. However, further studies are required to validate these findings.

## 1. Introduction

Radical esophagectomy for esophageal cancer is a highly invasive surgical procedure [1,2,3]. Therefore, minimally invasive procedures, such as thoracoscopic and robotic esophagectomy, have developed to reduce surgical trauma and postoperative respiratory complications [4,5]. Mediastinoscopic esophagectomy, which avoids thoracotomy and single-lung ventilation, has received increasing attention as a minimally invasive procedure, particularly in Japan and other East Asian countries [5,6,7,8]. This technique generally comprises transcervical and transhiatal phases. Orringer and Sloan first reported transhiatal esophagectomy without a transthoracic approach [9]. Although this technique avoids thoracotomy and one-lung ventilation, it involves blunt dissection during transcervical and transhiatal approaches and does not allow for mediastinal lymphadenectomy. Subsequently, Bumm et al. [10] and Tangoku et al. [11] reported that mediastinoscopic esophagectomy with mediastinal lymph node dissection can be safely performed using specialized equipment during the cervical approach. These procedures are alternative minimally invasive techniques that avoid the transthoracic approach and reduce the incidence of pulmonary complications. However, insufficient mediastinal visibility and a narrow surgical field make it a less radical technique.

We initially incorporated the transhiatal and cervical approaches as complementary procedures to reduce the duration of the thoracic phase. Subsequently, laparoscopic techniques gradually expanded the scope of cervical and transhiatal dissection and lymphadenectomy, allowing non-thoracotomy esophagectomy with radical lymph node dissection. This approach was covered by Japan’s national health insurance in 2018 as mediastinoscopic esophagectomy. We previously reported radical mediastinoscopic esophagectomy for esophageal cancer using the left cervical approach with single-port and transhiatal approach with the hand-assisted laparoscopic surgery (HALS) technique [12,13]. The HALS technique is commonly used during the abdominal phase of esophageal cancer surgery to reduce operative time and facilitate safe manipulation of the gastric tube [14]. During the transhiatal phase of mediastinoscopic esophagectomy, insertion of the surgeon’s left hand during HALS offers several advantages, such as stabilization of long surgical instruments and maintenance of a stable surgical field through liver retraction [15]. These advantages contribute to shorter operative times and greater procedural stability. Furthermore, the small epigastric incision created for insertion of the surgeon’s left hand can be effectively utilized for retrosternal reconstruction. However, the epigastric incision may cause postoperative pain, and concerns remain regarding whether HALS permits meticulous abdominal lymph node dissection.

The usefulness of HALS and laparoscopic surgery in the abdominal phase of transthoracic minimally invasive esophagectomy has been reported. Several comparative studies have shown that HALS has better perioperative outcomes than open laparotomy [16,17,18]. HALS was associated with less intraoperative blood loss, a lower systemic inflammatory response, and a lower incidence of pneumonia, whereas operative time and the number of harvested lymph nodes were comparable between the two approaches. It suggests that minimizing abdominal wall injury may contribute to less invasiveness and improved postoperative respiratory recovery. Importantly, available evidence has not supported concerns about the oncological adequacy of HALS. However, no significant differences in overall survival or abdominal recurrence-free survival were reported between HALS and open surgery, suggesting comparable oncological safety and local control [16,18]. On the other hand, several reports have compared laparoscopic surgery with HALS in the abdominal phase of minimally invasive esophagectomy, particularly for gastric mobilization and abdominal lymph node dissection [14,19,20]. Although a larger number of abdominal lymph nodes were harvested in laparoscopic surgery, the operative time was longer, and short-term outcomes were similar between the two groups. Yukawa et al. reported that a purely laparoscopic approach reduced the incidence of postoperative pneumonia compared with HALS [19]; however, other studies found no significant differences in postoperative complications or survival outcomes. Thus, although the laparoscopic approach may improve postoperative recovery and is both feasible and oncologically acceptable, HALS also represents a practical, less invasive approach without compromising surgical quality.

After accumulating sufficient clinical experience with the HALS technique and refining the surgical procedure, we established a totally laparoscopic transhiatal approach (Lap) for mediastinoscopic esophagectomy. We had been performing the HALS technique before 2016 [8] and introduced the laparoscopic technique in 2018. Since 2018, both techniques have been performed concurrently. For this study, we analyzed cases from 2020 onwards, after both techniques had been standardized. Although both techniques are based on similar concepts, they differ slightly in terms of surgical procedures and safety assurance. Therefore, in the present study, we describe the procedures and utility of the totally laparoscopic transhiatal technique used in mediastinoscopic esophagectomy and compare its surgical outcomes with those of HALS.

## 2. Materials and Methods

### 2.1. Patients and Characteristics

We retrospectively reviewed patients who underwent radical mediastinoscopic esophagectomy and gastric tube reconstruction for esophageal cancer using left cervical and transhiatal approaches at the Division of Digestive Surgery, Department of Surgery, Kyoto Prefectural University of Medicine, between January 2020 and June 2026. A total of 262 patients were reviewed; HALS was performed in 202 patients and laparoscopy (Lap) in 60 patients. The patients’ characteristics are presented in Table 1. Tumor progression was evaluated according to the Japanese Classification of Esophageal Cancer, 12th edition [21]. All patients were treated by three highly skilled surgeons and informed consent was obtained from each participant.

### 2.2. Surgical Procedures

Except for the transhiatal approach, the surgical procedures were similar between the groups and based on previously reported methods [8,13,15]. We briefly outline both approaches and their procedures. The patient was placed in a supine position with the legs abducted. General anesthesia was administered with a single-lumen endotracheal tube for bilateral lung ventilation.

#### 2.2.1. Cervical Phase

During the cervical approach, a 4 cm left collar incision was made, and a Lap-Protector (Hakko, Tokyo, Japan) was placed in the cervical wound and attached with an EZ access (Hakko) to establish the surgical working space. Two 5 mm trocars and a 5 mm AirSeal port (CONMED, FL, USA) were inserted through the access device. Continuous intraoperative monitoring of the left recurrent laryngeal nerve was used in all cases during the cervical procedure to facilitate safe dissection and minimize the risk of nerve injury. Short-acting muscle relaxants were administered only during induction of anesthesia and were withheld during the cervical phase to allow reliable identification and continuous monitoring of the recurrent laryngeal nerve. Muscular blockade was resumed at the beginning of the abdominal phase, according to the standard anesthetic protocol. Adequate exposure of the cervical operative field was achieved using a 5 mm retractor, the Jumbo Hook (Midorijasugiura, Tokyo, Japan), which was used to provide gentle and stable traction on the surrounding tissues. Esophageal mobilization and dissection were performed under laparoscopic visualization using the LigaSure Maryland jaw sealer (Medtronic, Tokyo, Japana), with careful attention to the surrounding structures, particularly the recurrent laryngeal nerve. Lymphadenectomy along the left recurrent laryngeal nerve was performed using scissors rather than electrocautery or a vessel-sealing device to minimize the potential for thermal injury during lymph node clearance.

#### 2.2.2. Abdominal and Transhiatal Phase

The choice between HALS and laparoscopy during the transhiatal phase was based on the surgeon’s preference, taking into account feasibility, cancer progression, and operative time. When the surgical procedure or exposure of the operative field was deemed difficult, or when precise lymph node dissection was considered difficult to achieve, the procedure was converted to HALS. Furthermore, when the preceding cervical approach was time-consuming or a non-curative resection was anticipated, the HALS procedure was selected to shorten operative time.

**Figure 1:**
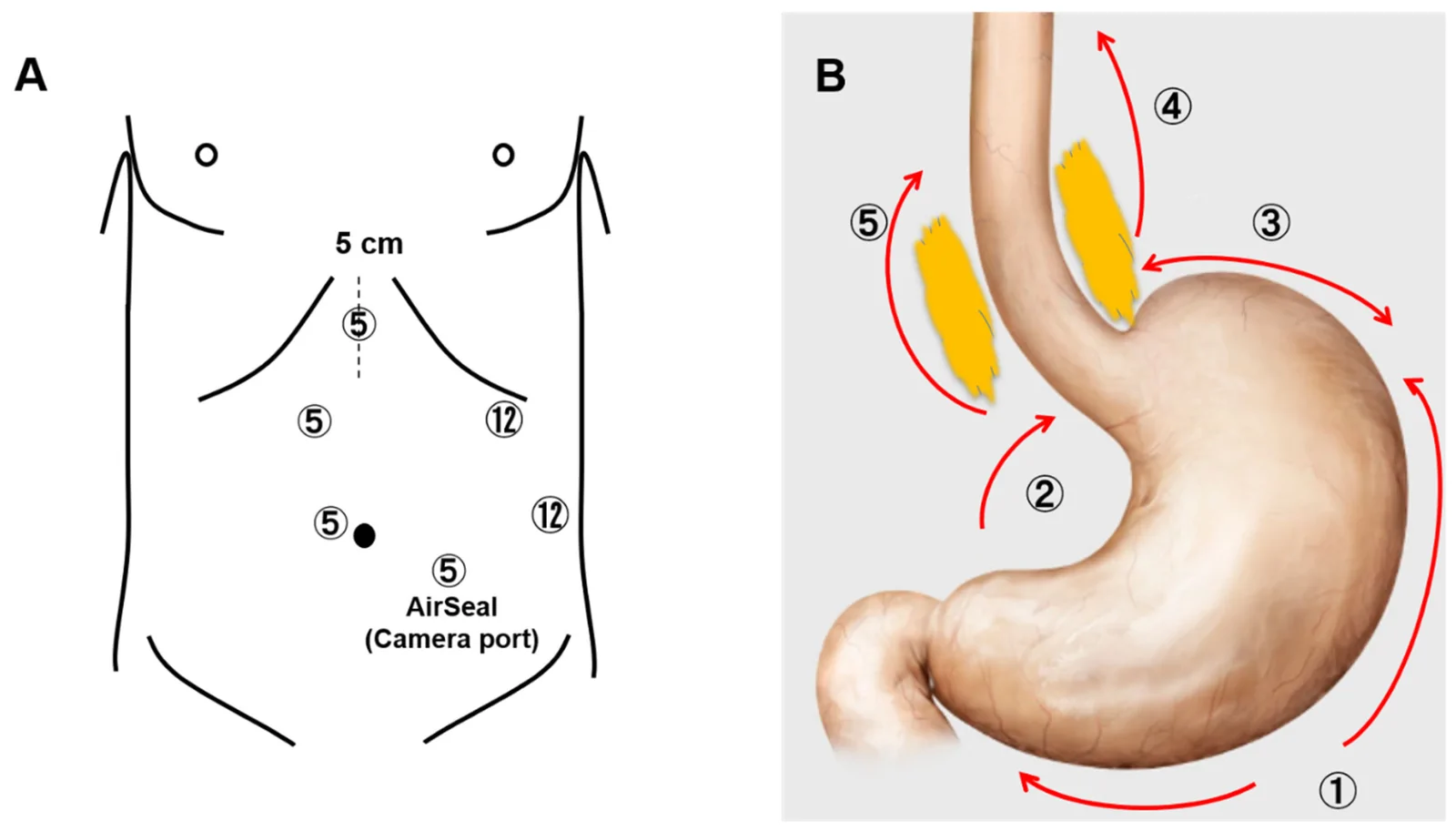
(**A**) Abdominal port placement for the laparoscopic transhiatal approach during mediastinoscopic esophagectomy. ⑤ and ⑫ indicate the 5 mm and 12 mm port, respectively. (**B**) Diagram of the surgical procedure extending from the area around the stomach to the lower mediastinum. The numbers indicate the order of management; the greater curvature of the stomach and the left gastroepiploic artery (①), the superior border of the pancreas (②), the gastrosplenic ligament (③), left side of the esophagus (④), and right side of the esophagus (⑤). The yellow color along the esophagus indicates the dissected fatty tissue on both sides of the esophagus.

Port placement for the laparoscopic transhiatal approach is shown in Figure 1A. During HALS, Lap Disc (Hakko) was placed in the 7 cm upper midline epigastric incision to establish the hand-assisted access. In both HALS and laparoscopic approaches, esophageal mobilization, mediastinal dissection, and lymphadenectomy were performed under laparoscopic visualization using the LigaSure device. During these procedures, the assistant provided stable exposure of the operative field using two specialized long retractors (Umihira, Kyoto, Japan), facilitating adequate visualization and safe dissection around the esophagus and surrounding structures. Lower mediastinal and abdominal lymph nodes at stations No. 108, 110, 112, 1, 2, 3, 7, 9, and 11p were dissected in all cases, while station No. 8a was dissected in 7 patients in the HALS group and 12 patients in the Lap group. In both groups, except for two patients, lymph node station No. 8a was dissected in patients with tumors located in the Jz region.

Following completion of the esophageal mobilization and lymphadenectomy, reconstruction was performed using a gastric tube through the retrosternal route. An enteral feeding tube was subsequently placed into the jejunum through the pyloric ring to provide postoperative nutritional support. The reconstruction procedure and feeding tube placement were performed in a similar manner in both the HALS and laparoscopic groups.

#### 2.2.3. Mediastinal Lymph Node Dissection

Typically, lymph node dissection of the upper and middle mediastinum was initially performed through the cervical approach, followed by dissection of the abdominal and lower mediastinal lymph nodes through the abdominal and transhiatal approaches. This sequential approach enabled systematic lymphadenectomy without transthoracic manipulation. When adequate dissection of the middle mediastinum could not be achieved through the cervical approach because of limited visualization or restricted instrument mobility, the dissection was supplemented or completed through the transhiatal approach. In particular, lymph node station 109 L, which is located along the left main bronchus and can be difficult to access from the cervical field, was selectively dissected through the transhiatal approach [12]. Depending on the extent of exposure and the anatomical conditions, a complete dissection of the middle mediastinal lymph nodes was achieved using cervical and transhiatal approaches as appropriate.

### 2.3. Statistical Analysis

Statistical analyses were performed using Fisher’s exact test and the unpaired *t*-test. Differences were considered statistically significant when the *p*-value was less than 0.05. All analyses were performed using statistical software (JMP Student Edition 19; SAS Institute Inc., Cary, NC, USA).

## 3. Results

### 3.1. Surgical Procedures in Laparoscopy

**Figure 2:**
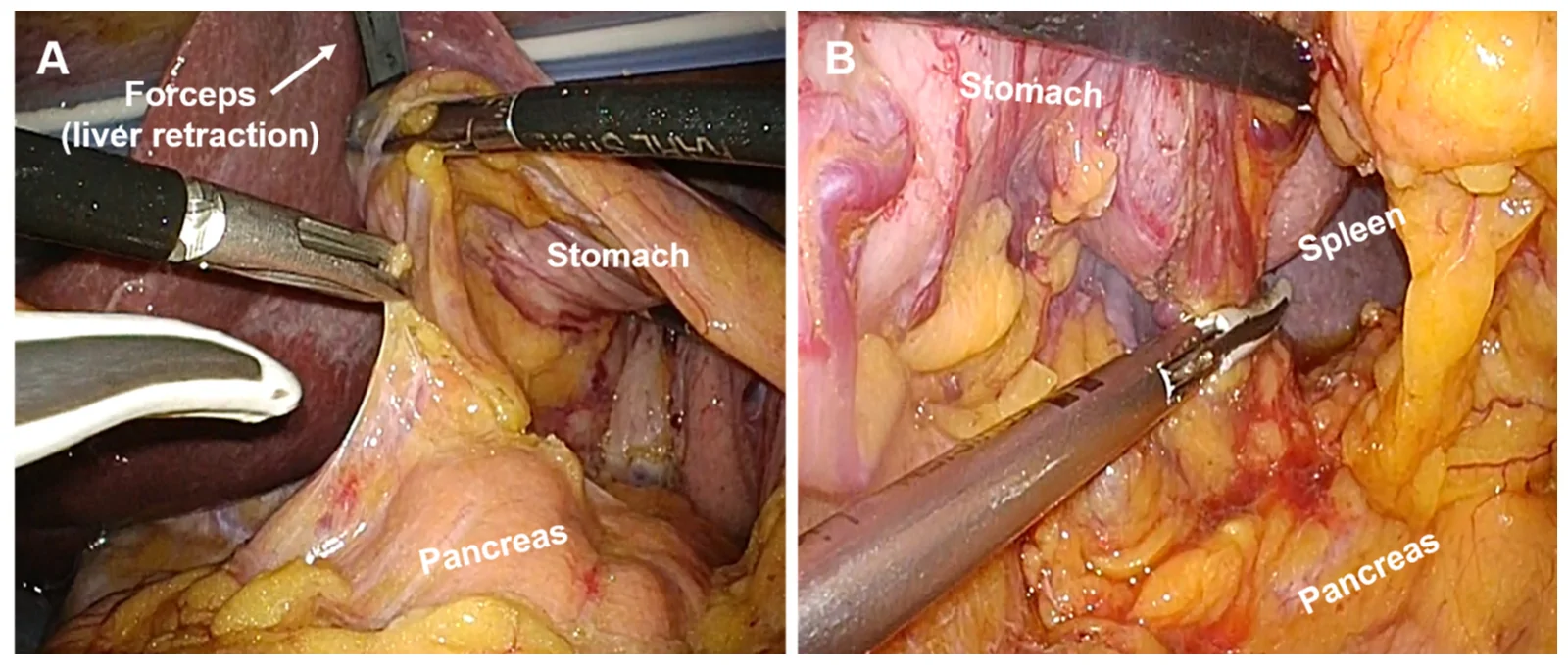
(**A**) Dissection and mobilization along the superior border of the pancreas from the side of the lesser curvature after the dissection of the left gastroepiploic artery along the greater curvature. Liver retraction forceps were inserted through an upper abdominal port to grasp the lesser curvature on the inferior surface of the liver. (**B**) Management of the gastrosplenic ligament.

Most procedures and instruments used in the laparoscopic transhiatal approach were identical to those used in the transhiatal approach during HALS [13,15]. A 5 mm port instrument manipulated with the surgeon’s left hand was placed slightly superior and to the right of the umbilicus (Figure 1A). The epigastric incision was reduced to approximately 5 cm and used for gastric tube reconstruction via the retrosternal route. During the laparoscopic procedure, a lap-disc and a 5 mm port were placed, and forceps were used to grasp the lesser curvature on the inferior surface of the liver to facilitate liver retraction (Figure 2A).

#### 3.1.1. Abdominal Phase

The procedure for lymph node dissection partially differed from that used in HALS (Figure 1B). After management of the left gastroepiploic artery along the greater curvature of the stomach, gastric mobilization and lymph node dissection along the superior border of the pancreas were initiated from the side of the lesser curvature (Figure 2A). This dissection adequately mobilized the stomach and facilitated subsequent access to the lymphatic tissue along the upper greater curvature. Subsequently, the gastrosplenic ligament was managed, and the dissection of the associated abdominal lymphatic tissue was completed (Figure 2B). These sequential steps safely achieved sufficient mobilization of the stomach and abdominal lymph node dissection, thereby creating an adequate operative field for the subsequent transhiatal approach.

#### 3.1.2. Transhiatal Phase

**Figure 3:**
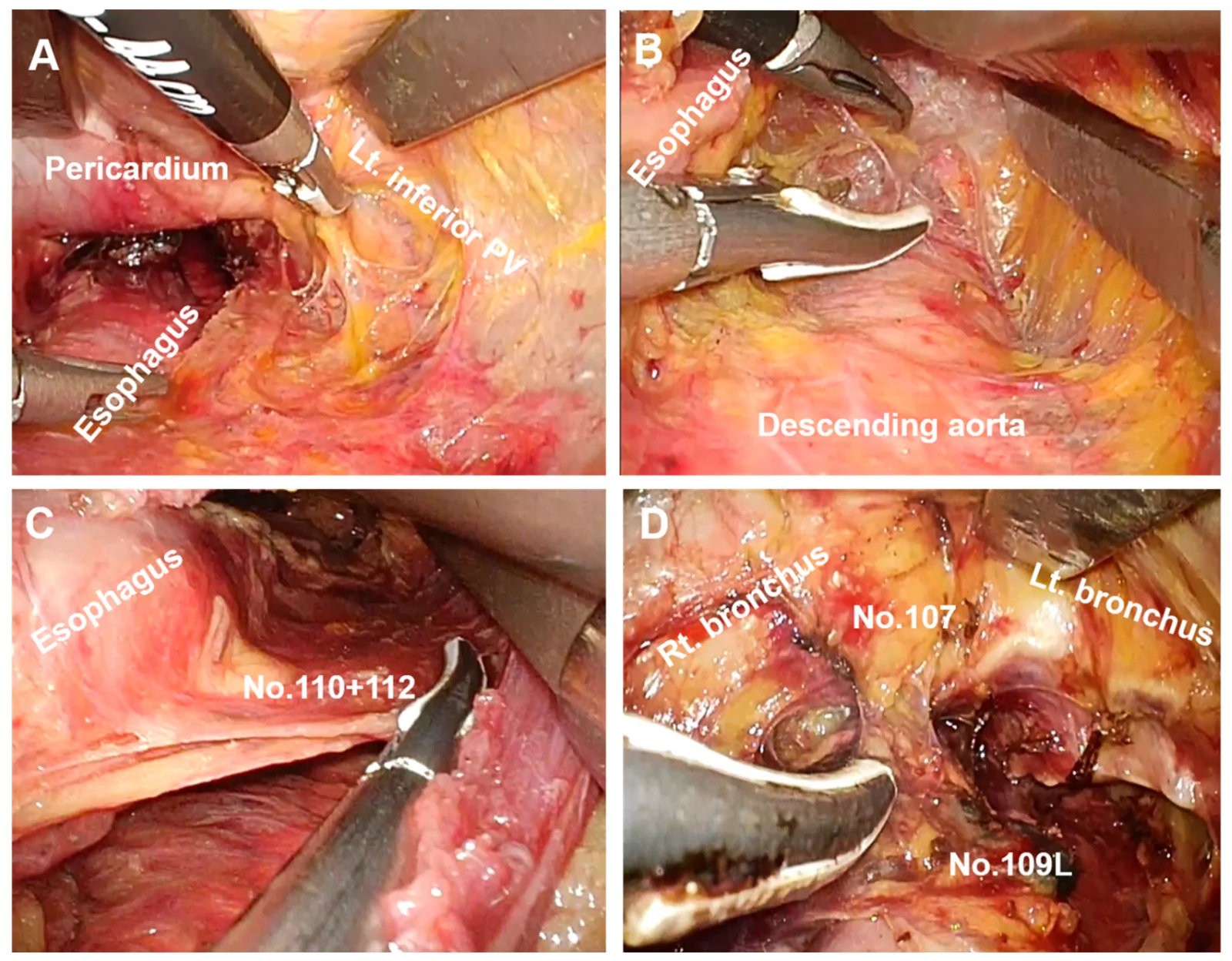
(**A**–**C**) Dissection of the lower mediastinum during the transhiatal approach. Dissection of the ventral side of the esophagus along the pericardium (**A**). Dissection of the dorsal side of the esophagus along the descending aorta (**B**). Lymph node dissection on the left side of the esophagus along the left mediastinal pleura (**C**). (**D**) Lymph node dissection at the tracheal bifurcation in the middle mediastinum. PV, pulmonary vein; Rt, right; Lt, left.

During the transhiatal approach, the ventral (Figure 3A) and dorsal sides (Figure 3B) of the esophagus were carefully dissected along the pericardium and descending aorta to connect with the plane dissected through the cervical approach. This procedure allows the fatty tissue containing mediastinal lymph nodes to be preserved on both sides of the esophagus, remaining continuous with the mediastinal pleura. Subsequently, the fatty tissue was dissected from the bilateral mediastinal pleura (Figure 3C), completing the en bloc mediastinal lymph node dissection up to the plane previously dissected through the cervical approach or to the level of the tracheal bifurcation (Figure 3D). These procedures provided adequate mobilization of the esophagus and surrounding lymphatic tissue while avoiding direct injury to the mediastinal pleura.

### 3.2. Comparison of Surgical Outcomes Between HALS and Lap

The surgical outcomes of both procedures are presented in Table 1. Patients in the Lap group had a significantly lower BMI. Histological types and the frequency of NAC were almost similar. There were no significant differences in blood loss and total operative time between the two techniques; however, the operative time for the transhiatal procedure tended to be longer in the Lap group (*p* = 0.09). Middle mediastinal lymphadenectomy was performed through the cervical and transhiatal approaches in both techniques (43% vs. 35%, *p* = 0.28). The total number of dissected lymph nodes, excluding the upper mediastinal and cervical lymph nodes, was similar between the groups. However, the number of dissected abdominal lymph nodes was significantly higher in the Lap group than in the HALS group (HALS vs. Lap; 15.7 vs. 17.9, *p* = 0.04). Clinicopathological characteristics, including tumor location, size, and progression, and postoperative complications did not differ between the two techniques. The R0 resection rate was higher and the hospital stay was shorter in the Lap group, but the differences were not statistically significant.

In the present study, laparoscopy was converted to HALS in two cases: one because a large lower esophageal tumor made surgical field exposure and forceps manipulation difficult, and the other because body habitus limited access to the middle mediastinum. Nevertheless, laparoscopic gastric mobilization and lymph node dissection were completed without complications in all cases, and no mortality was observed in the Lap group.

## 4. Discussion

In recent years, laparoscopic techniques have become widely used in minimally invasive gastrointestinal surgeries. However, the HALS technique remains relatively common during the abdominal phase of esophageal cancer surgery in Japan because it reduces operative time and permits gentle handling of the gastric tube [14,19,20]. HALS is also effective for the transhiatal approach during mediastinoscopic esophagectomy because it improves visualization through liver retraction and enhances instrument stability [15,22,23]. However, the larger incision adversely affects cosmetic outcomes and may increase wound pain or postoperative complications.

Briez et al. [24] conducted the MIRO trial, a multicenter randomized phase III trial that compared fully open esophagectomy with laparoscopically assisted (hybrid) esophagectomy. They hypothesized that the laparoscopic abdominal approach in esophageal cancer surgery would decrease the major postoperative complication rate owing to reduced surgical trauma. The hybrid group had significantly fewer major intraoperative and postoperative complications (36% vs. 64%, *p* < 0.001), and major pulmonary complications, in particular, decreased (18% vs. 30%) [25]. Although the five-year overall and disease-free survival rates were higher in the hybrid group, these differences were not statistically significant. These results indicated that minimally invasive abdominal procedures may reduce the risk of postoperative complications such as pneumonia. However, the difference between HALS and laparoscopic surgery remains a matter of debate.

We initially reported mediastinoscopic esophagectomy using the HALS technique [12]; subsequently, several studies reported the use of laparoscopic surgery for the transhiatal approach [26,27,28,29]. Daiko et al. reported that laparoscopic surgery was as safe as open surgery and permitted more effective lymph node dissection during minimally invasive esophagectomy [30]. They also used this laparoscopic technique for the transhiatal approach during mediastinoscopic esophagectomy [31]. Although surgical techniques vary across institutions, the middle mediastinum is frequently dissected through the cervical approach, whereas only the lower mediastinum is dissected through the transhiatal approach [26,27]. Complete middle mediastinum dissection in the transhiatal approach requires specialized forceps and technical modifications to ensure adequate visualization; consequently, the laparoscopic approach is more challenging than HALS [12,15].

Squamous cell carcinoma is the primary histological type of esophageal cancer in Japan and East Asia; therefore, abdominal lymphadenectomy focuses on the cardia and the left side of the superior border of the pancreas. In contrast, lymphadenectomy on the right side of the pancreas is important in esophageal adenocarcinoma and esophagogastric junction cancer [32]. During HALS, the surgeon’s left hand may interfere with lymph node dissection along the superior border of the pancreas, potentially compromising the quality of the dissection. The number of abdominal lymph nodes dissected was higher in the laparoscopy group than in the HALS group (Table 1), suggesting that this technique may be particularly useful in cases requiring more precise abdominal lymph node dissection. A more detailed analysis of the number of lymph nodes dissected at each station revealed that the number of nodes dissected at stations No. 3 + 9 was significantly higher in the Lap group (Supplementary Table S1). Given that station No. 3 can be dissected relatively easily using either approach, station No. 9, particularly the nodes located dorsal to the superior border of the pancreas, may be dissected effectively. However, patients in the Lap group had a significantly lower BMI than those in the HALS group (Table 1), which may have affected the number of dissected lymph nodes or R0 resection rate. Further studies involving a larger number of cases are required.

The surgical procedures differ slightly between HALS and laparoscopy. During HALS, the surgeon can easily manipulate the stomach and retract the liver with the left hand, allowing for continuous dissection of the mediastinum after management of the greater curvature of the stomach [13,15]. The ventral and dorsal sides of the esophagus were fully dissected to connect with the dissection layer performed through the cervical approach. The fatty tissue on the left side of the esophagus was dissected from the left mediastinal pleura, completing the left side mediastinal lymph node dissection up to the plane dissected through the cervical approach. Subsequently, mobilization and lymph node dissection were performed along the lesser curvature, extending from the superior border of the pancreas to the right side of the esophagus. However, gastric manipulation during laparoscopy is often more difficult than that during HALS. Therefore, we first completed gastric mobilization and lymph node dissection to ensure sufficient mobility before proceeding to mediastinal dissection (Figure 1B). Furthermore, to minimize bleeding and strain on the gastric tube, the gastrosplenic ligament along the greater curvature was managed after dissection of the left gastroepiploic artery and gastropancreatic ligament (Figure 2B).

During laparoscopy, the port for instruments manipulated with the surgeon’s left hand was placed closer to the midline compared to standard gastrectomy to improve accessibility to the mediastinum (Figure 1A). However, placing the port too close to the midline compromises instrument maneuverability along the superior border of the pancreas. Typically, the surgeon’s left-hand port should be placed closer to the midline, to the right of the falciform ligament, so as to avoid interference from this ligament. Then, the surgeon’s right-hand port should be placed further to the left than the left-hand port. In the present study, laparoscopy was converted to HALS in two cases because of the difficulties in instrument manipulation or limited access to the middle mediastinum. Therefore, port placement should be adjusted according to the patient’s body habitus, tumor location, and tumor extent.

This study has some limitations. This was a retrospective, single-center, non-randomized study with a small sample size. Published reports demonstrate that transhiatal mediastinoscopic esophagectomy is not a standardized procedure but encompasses various techniques [22,23,26,27,28,29]. Although port placement and surgical procedures vary among institutions, laparoscopy will be effective when lymph node dissection along the superior border of the pancreas is important. However, in the present study, the choice between HALS and Lap during the transhiatal phase was based on the surgeon’s preference, taking into account feasibility, cancer progression, and operative time. In this context, although prior surgeries and tumor progression did not differ between the two groups, BMI was significantly lower in the Lap group, suggesting possible surgeon selection bias. It will be necessary to consider the criteria for selecting suitable cases in the future. The utility of robotic surgery during the transhiatal phase was not evaluated in this study. Robotic mediastinal esophagectomy may facilitate instrument manipulation within the narrow mediastinum and along the superior border of the pancreas [33,34]. We have also performed robotic transhiatal esophagectomy; however, the number of cases is limited, and the procedure has not yet been standardized. Preserving the mediastinal pleura may be difficult during robotic surgery. Further refinement and standardization of the surgical technique are needed, including the use of an additional retractor to maintain a clear operative field.

## 5. Conclusions

The laparoscopic procedure was performed safely, and the number of abdominal lymph nodes dissected during laparoscopy was not inferior to that during HALS. Although further study is needed, this approach may be useful in cases requiring more precise abdominal lymph node dissection.

## Figures and Tables

**Table 1:** Comparison of surgery-related factors between HALS and Lap groups.

Subjects	Total (*n* = 262)	HALS (*n* = 202)	Lap (*n* = 60)	*p* Value
BMI (average (range))		21.6 (13.9–28.4)	19.4 (13.5–28.4)	<0.01
Histological type	SCC/Adenocarcinoma	168/34	52/8	0.52
NAC	+/−	119/83	31/29	0.32
Blood loss (g)		150	137	0.53
Operative Time (min)	Total	346	338	0.37
	Hiatal approach	102	110	0.09
Middle mediastinal	Cervical/Both approach/None	88/86/28	33/21/6	0.28
lymphadenectomy				
Number of	Total	26.8	29.4	0.1
dissected lymph node	Middle Med.	8.3	7.8	0.54
(without upper Med.)	Lower Med.	4.2	4.5	0.55
	Abdominal	15.7	17.9	0.04
Location	Jz/Lt/Mt/Ut	13/64/88/37	9/19/25/7	0.15
Size	(mm)	41.6	42.8	0.79
pT	0/1/2/3/4	14/81/17/82/8	4/22/5/24/5	0.75
pN	0/1/2/3	100/53/37/12	27/17/8/8	0.24
pStage	0/I/II/III/IV	9/55/59/60/19	4/15/16/21/4	0.83
Residual tumor	R0/1/2	164/18/20	56/2/2	0.08
Postoperative complications				
Pulmonary complications	+/−	32/170	4/56	0.07
Anastomotic leakage	+/−	28/174	9/51	0.82
SSI (cervical wound)	+/−	18/184	4/56	0.58
SSI (abdominal wound)	+/−	14/180	3/57	0.55
Pleural effusion	+	11	4	
Ileus	+	1	1	
Postoperative hospital stay	(days)	32.6	29	0.13
Mortality	30 days	1 (Aspiration)	0	
	90 days	1 (Sepsis)	0	

BMI; body mass index, NAC; neoadjuvant chemotherapy, SCC; squamous cell carcinoma; Med.; mediastinal, SSI; surgical site infection.

## Data Availability

The data presented in this study are available on request from the corresponding author due to privacy.

## Supplementary Materials

The following supporting information can be downloaded at https://www.mdpi.com/article/10.3390/cancers18193236/s1: Table S1: The number of abdominal lymph nodes dissected at each station.

## Abbreviations

The following abbreviations are used in this manuscript:

HALS	hand-assisted laparoscopic surgery
Lap	laparoscopy
BMI	body mass index
NAC	neoadjuvant chemotherapy
SCC	squamous cell carcinoma
Med.	mediastinal
SSI	surgical site infection
