# Supplementary material for: A Totally Laparoscopic Transhiatal Approach for Esophageal Cancer During Mediastinoscopic Esophagectomy
Source: Cancers (Basel). 2026 Oct 8;18(19):3236. doi: 10.3390/cancers18193236 (PMC13664125; doi:10.3390/cancers18193236)
Supplement: Supplementary file 1 [file cancers-18-03236-s001.zip › cancers-4567099-supplementary.pdf]

Supplementary Table S1. The number of abdominal lymph nodes dissected at each station

	HALS (n= 202)	Lap (n= 60)	p value
No. 1	2.7	2.8	0.83
No.2	3.5	3.7	0.6
No.3+ 9	4.9	6.5	$p < 0.05$
No.7+ 11p	4.7	4.8	0.95
No. 8a	2.7	2.8	0.86
